# Identification of homologous GluN subunits variants accelerates *GRIN* variants stratification

**DOI:** 10.3389/fncel.2022.998719

**Published:** 2022-12-23

**Authors:** Ana Santos-Gómez, Adrián García-Recio, Federico Miguez-Cabello, David Soto, Xavier Altafaj, Mireia Olivella

**Affiliations:** ^1^Neurophysiology Laboratory, Department of Biomedicine, Faculty of Medicine and Health Sciences, Institute of Neurosciences, University of Barcelona, Barcelona, Spain; ^2^August Pi i Sunyer Biomedical Research Institute (IDIBAPS), Barcelona, Spain; ^3^Bioinfomatics and Medical Statistics Group, University of Vic—Central University of Catalonia, Barcelona, Spain

**Keywords:** NMDA receptor, GRIN, epilepsy, intellectual disability, mutation predictor

## Abstract

The clinical spectrum of *GRIN*-related neurodevelopmental disorders (GRD) results from gene- and variant-dependent primary alterations of the NMDA receptor, disturbing glutamatergic neurotransmission. Despite *GRIN* gene variants’ functional annotations being dually critical for stratification and precision medicine design, genetically diagnosed pathogenic *GRIN* variants currently outnumber their relative functional annotations. Based on high-resolution crystal 3D models and topological domains conservation between GluN1, GluN2A, and GluN2B subunits of the NMDAR, we have generated GluN1-GluN2A-GluN2B subunits structural superimposition model to find equivalent positions between GluN subunits. We have developed a *GRIN* structural algorithm that predicts functional changes in the equivalent structural positions in other GluN subunits. GRIN structural algorithm was computationally evaluated to the full *GRIN* missense variants repertoire, consisting of 4,525 variants. The analysis of this structure-based model revealed an absolute predictive power for GluN1, GluN2A, and GluN2B subunits, both in terms of pathogenicity-association (benign vs. pathogenic variants) and functional impact (loss-of-function, benign, gain-of-function). Further, we validated this computational algorithm experimentally, using an *in silico* library of GluN2B-equivalent GluN2A artificial variants, designed from pathogenic GluN2B variants. Thus, the implementation of the GRIN structural algorithm allows to computationally predict the pathogenicity and functional annotations of *GRIN* variants, resulting in the duplication of pathogenic *GRIN* variants assignment, reduction by 30% of *GRIN* variants with uncertain significance, and increase by 70% of functionally annotated *GRIN* variants. Finally, GRIN structural algorithm has been implemented into *GRIN* variants Database (http://lmc.uab.es/grindb), providing a computational tool that accelerates *GRIN* missense variants stratification, contributing to clinical therapeutic decisions for this neurodevelopmental disorder.

## 1 Introduction

*GRIN*-related neurodevelopmental disorders (GRDs) constitute a group of rare genetic diseases with a broad clinical spectrum including intellectual disability, epilepsy, movement disorders, development delay, autism spectrum disorder, and schizophrenia (Lesca et al., 2013; Lemke et al., 2014, 2016; Burnashev and Szepetowski, 2015). The primary cause of GRD is the presence of *de novo* *GRIN* variants—with almost exclusively autosomal dominant inheritance pattern—that results in the presence of dysfunctional GluN subunits of the N-methyl D-Aspartate receptor (NMDAR). The NMDAR belongs to the glutamate ionotropic receptors family and plays a pivotal role in neuronal development, synaptic plasticity, and neuron survival (Paoletti et al., 2013). NMDA receptors are composed of two obligatory GluN1 subunits, and a combination of two additional GluN subunits (GluN2A-D, GluN3A,B) encoded by *GRIN1*, *GRIN2A-D*, and *GRIN3A,B*, respectively (Paoletti et al., 2013). The NMDA receptor tetrameric architecture consists of an Amino Terminal Domain (ATD), a Ligand Binding Domain (LBD), a Transmembrane Domain (TMD), and a large Carboxy Terminal Domain (CTD, with an unsolved structure). Concurrent binding of glycine to the GluN1 subunit and glutamate to the GluN2 subunit is required for NMDAR activation (Furukawa et al., 2005) and recent studies shed light on the conformational changes due to receptor activation and inhibition (Chou et al., 2020; Wang et al., 2021).

Since the identification of GRD genetic etiology (Endele et al., 2010; Tarabeux et al., 2011; de Ligt et al., 2012), the number of *GRIN* variants has exponentially grown, being currently represented by 4,525 reported unique variants^1^ (July 2021; García-Recio et al., 2021a). Despite some variants affecting *GRIN2C*, *GRIN2D*, *GRIN3A*, and *GRIN3B* genes have been described, the vast majority (95%) of GRD-associated variants are associated with *GRIN1*, *GRIN2A*, and *GRIN2B* genes. Genotype-phenotype association studies are partially accompanied by functional studies of *GRIN* variants gene products, allowing us to assign both their pathogenicity likelihood and their functional stratification. Currently, a total of 445 *GRIN* variants are considered pathogenic in patients with neurodevelopmental disorders, while 3,800 variants are classified as benign and 275 variants are considered to be of uncertain significance. Overall, the number of *GRIN* variants of certain significance annotation (i.e., genetic and experimental data supporting variant-associated NMDAR dysfunction) is still limited. The identification of novel *GRIN* variants in pediatric patients needs experimental validation, both to assign likely pathogenicity (i.e., evaluation of variant-related protein dysfunction) and to annotate the functional outcomes that are roughly classified into loss-of-function (LoF), gain-of-function (GoF) or complex effects. In turn, *GRIN* variants stratification is crucial to provide the molecular diagnosis supporting the selection of a given personalized therapeutic strategy option. Importantly, in the context of the temporal dimension of this neurodevelopmental disorder, accelerating *GRIN* variants annotation is crucial to early define precision therapeutic arms, either to rescue LoF [L-serine (Soto et al., 2019)] or GoF variants [memantine (Pierson et al., 2014), radiprodil (Mullier et al., 2017; Auvin et al., 2020), dextromethorphan].

The lack of high-throughput experimental methods for *GRIN* variants annotation and their intrinsic position-dependent phenotypic alterations result in a growing gap between *GRIN* variants’ genetic identification and their relative GluN subunits’ functional annotations. Together with numerous efforts to annotate *GRIN* variants, computational tools provide a powerful strategy to accelerate *GRIN* variants annotation. In this context, we recently released the *GRIN* variants Database (GRINdb; García-Recio et al., 2021a), comprehensively compiling available genetic, clinical and functional data related to *GRIN* variants. In addition to providing individual *GRIN* variants information with clinical outputs, *GRIN* variants Database analysis can accelerate *GRIN* variants annotation. In this regard, GRINdb revealed the subunit and domain variables defining GluN truncating variants’ pathogenicity (Santos-Gómez et al., 2021).

Based on GluN subunits’ structural conservation within the NMDAR tetramer, in this study, we have developed and experimentally validated -both *in silico* and *in vitro*- a structural computational algorithm that significantly extends the assignment of pathogenicity and functional parameters of non-annotated *GRIN* homologous variants. The algorithm has been implemented into the open access and most comprehensive *GRIN* variants Database, providing additional tools for pathogenicity and functional *GRIN* variants stratification, contributing to clinical decision-making.

## 2 Methods

### 2.1 Development of GRIN structural algorithm

GluN1, GluN2A, and GluN2B subunits Amino Terminal Domain (ATD), Ligand Binding Domain (LBD), and Transmembrane Domains (TM; see Supplementary Table 1 for detailed domain coordinates) were structurally superimposed using PyMOL 3 (DeLano, 2002). The residue ranges for each domain were determined using OPM (Lomize et al., 2012). The X-ray crystal structure of human GluN1/GluN2A diheteromeric NMDA receptor (PDB ID 6IRA; Zhang et al., 2018) was used to extract GluN1 and GluN2A structures, and the X-ray crystal structure of rat GluN1/GluN2B diheteromeric NMDA receptor (PDB ID 6WHR; Chou et al., 2020) was used to extract GluN2B structural model. Based on the structural sequence alignment, the alpha carbon root mean square deviation (RMSD) and sequence identity were computed for each subunit pair. The sequence alignment obtained from the structural superimposition of GluN subunits was used to identify equivalent positions between GluN1, GluN2A, and GluN2B subunits. For the structurally-unsolved regions, sequence alignment was used to identify equivalent positions. In summary, for a given GluN variant, the structural algorithm retrieves the corresponding homologous variant/s (same amino acid change in the same equivalent position) and the pathogenesis and functional annotation/s of the homologous variants are extrapolated to the given GluN variant.

### 2.2 Computational evaluation of GRIN structural algorithm

In order to evaluate the pathogenesis and functional annotations predictive power of the algorithm, all GluN variants’ pathogenicity (pathogenic, uncertain significance, or benign) and functional annotations (LoF/GoF/Complex) were retrieved from the *GRIN* variants Database^1^ (García-Recio et al., 2021a). *GRIN* variants Database is the most comprehensive *GRIN* variants repertoire, integrating pathogenesis assignments and functional annotations retrieved from Gnomad (Karczewski and Francioli, 2017), ClinVar, CFERV^2^, LOVD^3^, Uniprot (UniProt Consortium, 2019) databases, as well as from the bibliography. The pathogenicity and functional annotations for equivalent positions between GluN subunits were compared for homologous variants, i.e., same initial and final amino acid. Additionally, the annotations were also compared between equivalent GluN positions for: (i) different initial amino acids and the same final amino acid and for; and (ii) the same initial amino acid with different final amino acids.

### 2.3 Computational design and experimental validation of homologous GluN artificial variants

In order to experimentally validate the structural superimposition model, a collection of nine domain-representative GluN2A variants was designed using the structural algorithm, homologous to nine previously reported GluN2B pathogenic variants. Functional annotations were performed for the following pairs of *GRIN2B* pathogenic variants—*GRIN2A* *in silico-*designed variants: GluN2B p.(Gly459Arg)-GluN2A p.(Gly458Arg); GluN2B p.(Gly484Asp)-GluN2A p.(Gly483Asp); GluN2B p.(Thr532Ala)-GluN2A p.(Thr531Ala); GluN2B p.(Gly543Arg)-GluN2A p.(Gly542Arg); GluN2B p.(Gly689Sser)-GluN2A p.(Gly688Ser); GluN2B p.(Arg693Gly)-GluN2A p.(Arg692Gly), GluN2B p.(Gly820Ala)-GluN2A p.(Gly819Ala), GluN2B p.(Gly820Glu)-GluN2A p.(Gly819Glu), GluN2B p.(Met824Val)-GluN2A p.(Met823Val).

### 2.4 Plasmids

The expression plasmids encoding for wildtype rat HA-GluN1, GFP-GluN2A, and GFP-GluN2B subunits were kindly provided by Dr. Nakanishi and Dr. Vicini (Vicini et al., 1998), respectively. Nucleotide changes for the production of *GRIN* variants were achieved by oligonucleotide-directed mutagenesis, using the QuickChange II XL site-directed mutagenesis kit according to the manufacturer’s instructions (Stratagene), and verified by Sanger sequencing.

### 2.5 Cell culture and transfection

HEK-293T and COS-7 cell lines were obtained from the American Type Culture Collection and maintained at 37°C in Dulbecco’s modified Eagle’s medium (DMEM), supplemented with 10% fetal calf serum and antibiotics (100 units/ml penicillin and 100 mg/ml streptomycin) and D-2-amino-5-phosphonopentanoic acid (D-AP5, Abcam; 0.5–1 mM final concentrations, for HEK-293T and COS-7 cells, respectively) to prevent excitotoxicity. Transient expression of NMDARs in HEK-293T cells was achieved with polyethylenimine (PEI)-based transfection method, and NMDAR-mediated currents were recorded 24 h after transfection. COS-7 cells were transfected with Lipofectamine^TM^ 2000 (Invitrogen) following the manufacturer’s instructions, and cells were fixed 24 h post-transfection for further immunofluorescence analysis. Cells were transfected with equimolar amounts of GluN1 and GFP-GluN2A/GluN2B subunits (1:1) for immunofluorescence experiments, or with a 1:2 (GluN1:GluN2) ratio for electrophysiological recordings.

### 2.6 Immunofluorescence analysis

Transiently transfected COS-7 cells were washed in PBS and fixed with 4% paraformaldehyde. Surface expression of NMDARs was achieved by immunolabeling extracellular GFP (GFP cloned in-frame within GluN2 subunits ATD), incubating with anti-GFP antibody (Clontech) for 1 h at RT under non-permeabilizing conditions. After washing, cells were incubated with anti-rabbit IgG-Alexa555 secondary antibodies (Life Technologies, Carlsbad, CA, USA), for 1 h at RT. The total amount of GFP-tagged GluN subunits was detected by the GFP endogenous fluorescent signal emitted by GFP-GluN2A/GluN2B constructs. Coverslips were mounted in ProLong antifade mounting medium (Life Technologies) and images were acquired in a Nikon Eclipse 80i microscope (63×/1.4 N.A. immersion oil objective).

### 2.7 Electrophysiological recordings of NMDAR-mediated currents in HEK293T cells

Electrophysiological recordings were performed 24 h after transfection, perfusing the cells continuously with extracellular physiological bath solution (in mM): 140 NaCl, 5 KCl, 1 CaCl_2_, 10 glucose, and 10 HEPES, adjusted to pH 7.42 with NaOH. Glutamate (1 mM, Sigma-Aldrich) and glycine (50 microM; Tocris) were co-applied for 5 s by piezoelectric translation (P-601.30; Physik Instruments, Karlsruhe, Germany) of a theta-barrel application tool made from borosilicate glass (1.5 mm o.d.; Sutter Instruments) and the activated currents were recorded in the whole-cell configuration at a holding potential of −60 mV, acquired at 5 kHz and filtered at 2 kHz by means of Axopatch 200B amplifier, Digidata 1440A interface and pClamp10 software (Molecular Devices Corporation, San José, CA, USA). Electrodes with open-tip resistances of 2–4 MΩ were made from borosilicate glass (1.5 mm o.d., 0.86 mm i.d., Harvard Apparatus, Cambridge, MA, USA), pulled with a P-97 horizontal puller (Sutter Instruments) and filled with intracellular pipette solution containing (in mM): 140 CsCl, 5 EGTA, 4 Na_2_ATP, 0.1 Na_3_GTP, and 10 HEPES, adjusted to pH 7.25 with CsOH. Glutamate plus glycine-evoked currents were expressed as current density (-pA/pF; maximum current divided by input capacitance, as measured from the amplifier settings) to avoid differences due to surface area in the recorded cells. The kinetics of deactivation and desensitization of the NMDAR responses were determined by fitting the glutamate/glycine-evoked responses at V_m_= −60 mV to a double-exponential function in order to determine the weighted time constant (τ_w,des_):


$$\tau_{\text{w,des = }\tau_{\text{f}}}\left(\frac{{\text{A}}_{\text{f}}}{{\text{A}}_{\text{f+}}{\text{A}}_{\text{s}}}\right)+\tau_{\text{s}}\left(\frac{{}_{\text{s}}}{{\text{A}}_{\text{f}}+{\text{A}}_{s}}\right)$$

where A_f_ and τ_f_ correspond to the amplitude and time constant of the fast component of desensitization and A_s_ and τ_s_ are the amplitude and time constant of the slow component of desensitization.

### 2.8 Statistical analysis

Comparison between experimental groups was evaluated using Prism9 (GraphPad Software, Inc., San Diego, CA, USA), applying a One-Way Analysis of Variance (ANOVA) followed by a Bonferroni *post-hoc* test (cell surface expression experiments) or Mann-Whitney U-test (for electrophysiology experiments). Data are presented as the mean ± SEM from at least three independent experiments.

### 2.9 Assessment of GRIN structural algorithm predictive power

In order to evaluate the predictive power of GRIN structural algorithm, the algorithm was applied to all GluN variants (4,525) contained in the *GRIN* variants Database. Pathogenicity and functional annotations were predicted for non-annotated *GRIN* homologous variants.

### 2.10 Integration of GRIN structural algorithm into the GRIN variants database

*GRIN* structural algorithm was implemented into *GRIN* variants Database^1^. For a given GluN variant, the algorithm allows retrieval of all pathogenicity and functional annotations of homologous GluN variants (upon availability), using a user-friendly interface.

## 3 Results

### 3.1 Generation of GRIN structural algorithm

Pairwise structural alignment of human GluN1, GluN2A, and GluN2B subunit structures has been performed, delivering the identification of GluN subunits’ equivalent positions across the extracellular and transmembrane domains (Supplementary Figure 1). Quantitative analysis of GluN subunits’ structural alignment accuracy showed a high degree of structural conservation between GluN subunits’ domains. Indeed, root mean square deviation (RMSD) values revealed the structure conservation (ranging from very high to high) between GluN subunits (see Table 1). In terms of pairwise subunit sequence identity, the analysis indicated the presence of high sequence identity between GluN2A and GluN2B subunits and moderate conservation between GluN1-GluN2A or GluN1-GluN2B subunits. In terms of functional domains, the analysis showed a high sequence identity and structure conservation within the LBD and TMD, and to a lesser extent in the amino acid sequence of the ATD. The domain-specific structural conservation is coincident with the distribution of *GRIN* variants disease association. Indeed, the *GRIN* variant’s pathogenicity is more elevated in amino acids located within the LBD and TMD, while *GRIN* missense variants within ATD are variably associated with neurological conditions (García-Recio et al., 2021a). Although some particular NMDAR subdomains present a limited sequence identity, the structure conservation between GluN subunits (i.e., overall RMSD values) allows the generation of an accurate GluN subunits’ structural superimposition model, allowing the identification of precise equivalent positions between GluN subunits. This is in compliance with the reported higher structure conservation compared to sequence conservation in proteins (Rodionov and Blundell, 1998), which is particularly more pronounced in membrane proteins (Olivella et al., 2013).

**Table 1:** Amino acid sequence identity and root mean standard deviation (RMSD) between GluN subunits, based on pairwise structural alignment of GluN subunit topological domains.

		GluN2A-GluN1	GluN2B-GluN1	GluN2A-GluN2B
ATD	*Identical residues*	60 (out of 401)	48 (out of 402)	220 (out of 402)
	*Sequence identity*	14,96%	11,94%	54,73%
	*RMSD (ATD1)*	2.034	3.106	1.096
	*RMSD (ATD2)*	1.062	1.324	0.989
LBD	*Number of identical residues*	104 (out of 317)	99 (out of 317)	265 (out of 317)
	*Sequence identity*	32,81%	31,23%	83,60%
	*RMSD*	1.229	1.653	0.832
TMD	*Number of identical residues*	44 (out of 140)	46 (out of 140)	131 (out of 140)
	*Sequence identity*	31,65%	33,09%	92,86%
	RMSD	1.899	1.774	1.312

ATD, amino terminal domain; LBD, ligand binding domain; TMD, transmembrane domain.

### 3.2 Computational evaluation of GRIN structural algorithm

GRIN structural algorithm was applied to the full repertoire of annotated *GRIN* variants, retrieved from *GRIN* variants Database (GRINdb; García-Recio et al., 2021a). Pathogenicity and functional annotations for homologous *GRIN* variants were identified and extracted for comparison. Additionally, the comparison was performed for non-homologous equivalent positions.

#### Comparative analysis of GluN homologous variants annotations

**Figure 1:**
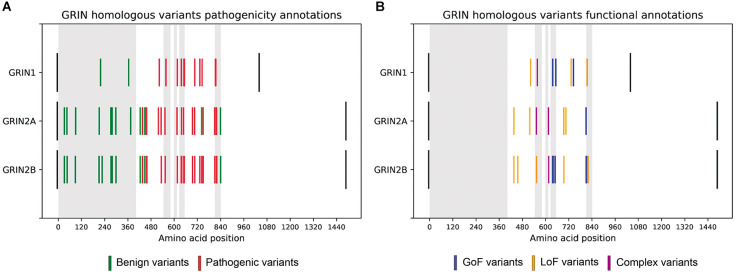
**(A)** Comparative analysis of *GRIN* homologous variants pathogenesis in *GRIN* variants Database. Disease-associated variants are colored in red and benign variants are colored in green. Black bars represent the initial and final amino acids of canonical GluN1, GluN2A, and GluN2B subunits. Gray rectangles represent the amino-terminal domain, and transmembrane domains (TM1, TM2, TM3, TM4, respectively). Homologous variants present the same pattern of pathogenesis across GluN1, GluN2A, and GluN2B subunits. **(B)** Functional annotations of *GRIN* homologous variants *in GRIN* variants Database. LoF variants are colored in yellow, while GoF variants are colored in blue.

First, 87 *GRIN* missense variants corresponding to 33 homologous variants, i.e., same amino acid in equivalent positions between subunits, were identified. For each pair or trio of homologous variants, qualitative disease association was compared. Variants with uncertain significance were discarded, resulting in 28 benign variants and 50 pathogenic filtered variants (see Supplementary Table 2, Figure 1A). Importantly, in terms of disease association, the comparison of these 78 variants showed a 100% coincidence, indicating complete disease-association pattern conservation across the annotated GluN1-GluN2A-GluN2B subunits missense variants.

The functional annotations for homologous variants were extracted for comparison, resulting in 8 pairs of homologous variants with available LoF/GoF/Complex functional classification (see Supplementary Table 2). The functional classification of these eight pairs of homologous variants was coincident (see Figure 1B), strongly supporting that pathogenicity and functional disturbances induced by homologous variants affecting ATD, LBD, and TMD domains of GluN1, GluN2A, and GluN2B are coincident.

#### Comparative analysis of non-homologous variants in equivalent positions

The pathogenesis and functional annotations of 27 variants of 13 equivalent positions with different initial amino acids and identical final amino acids were compared (see Supplementary Table 3). With the exception of GluN1 p.(Lys685Arg)-GluN2B p.(Pro687Arg), disease association of non-homologous variants in equivalent positions (12 pairs variants out of 13) was coincident. Amongst them, in terms of functional classification, the only pair with available functional annotations showed coincident functional outcomes. Second, the pathogenesis and functional annotations of 210 variants from 72 equivalent positions with the same initial amino acid and different final amino acids were compared (see Supplementary Table 4). From these variants, 59 pairs (out of 72) were coincident in terms of disease-association and functional annotations were coincident in 23 out of 30 pairs. Interestingly, some variants affecting the same initial residue mutated into different final residues revealed a differential pathological outcome. Indeed, GluN2B p.(Ala639Val) vs. GluN2B p.(Ala639Ser) comparison showed either a benign or pathogenic effect. These data suggest that besides residue position, the final amino acid’s intrinsic physicochemical properties strongly determine the structural, functional, and pathological impact. Overall, these findings indicate that non-homologous variants of *GRIN1*, *GRIN2A*, and *GRIN2B* genes can neither be used to extrapolate disease association nor functional annotations.

### 3.3 Experimental validation of GRIN structural algorithm

Upon the generation of the GluN subunits’ structural model, the algorithm’s predictive power was experimentally evaluated *in vitro*. Based on GRD patients-associated *GRIN2B* variants referred to our group, we conducted the functional annotation of *in silico*-designed *GRIN2A* (“artificial”) variants putatively equivalent and compared their functional outcomes to those from *GRIN2B* pathogenic variants. The artificial *GRIN2A* variants were selected to representatively cover *GRIN* variant vulnerable domains (e.g., prevalently associated with *GRIN de novo* pathogenic variants), e.g., localized at the glutamate binding domain (six variants) and the transmembrane domain (three variants). The comparative analysis of the functional impact of GluN2A-GluN2B homologous pairs was systematically performed in parallel, using mammalian heterologous expression systems. First, mutant NMDAR surface expression was assessed by immunofluorescence analysis in COS-7 cells transiently expressing di-heteromeric (GluN1wt)_2_-(GluN2Amut)_2_ or (GluN1wt)_2_-(GluN2Bmut)_2_, and showed surface trafficking patterns conservation between artificial GluN2A variants and equivalent GRD-associated GluN2B pairs (Supplementary Figure 2). Indeed, *GRIN2B* variants leading to mild or unaffected NMDAR surface trafficking were compared with their relative pairs, showing an overall conserved impact (Supplementary Figure 2). This pattern conservation was especially noticed in a pair of variants showing a drastic effect, e.g., abolishing mutant NMDARs surface trafficking (i.e., GluN2A p.(Gly458Arg)-GluN2B p.(Gly459Arg) pair).

**Figure 2:**
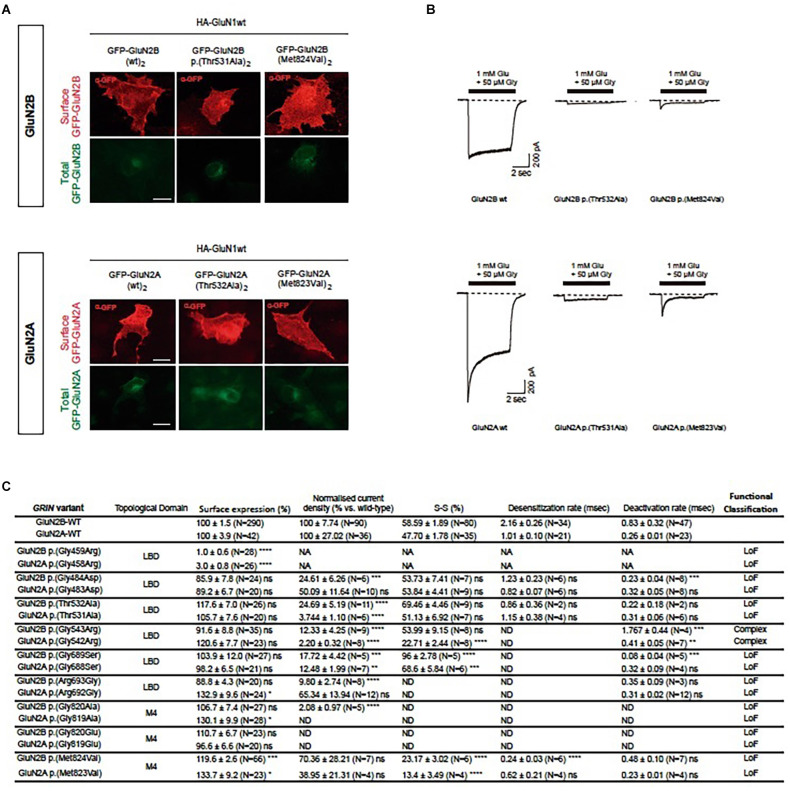
Comparative analysis of functional alterations in GluN2A-GluN2B predicted equivalent pairs. **(A)** Representative images of immunofluorescence detection of surface trafficking from artificial GluN2A variants-containing NMDARs (upper panels), designed upon respective patients-associated GluN2B variants (lower panels). Transfected COS-7 cells heterologously expressing GFP-GluN2A/B subunits (green channel, intracellular expression) were immunostained and surface expression of mutant GluN2A/B labeled (red channel). **(B)** Representative electrophysiological traces of NMDAR-mediated currents in HEK-293T cells transfected with GluN1 and GluN2A/2B pairs (upper panels: (GluN1wt)_2_-(GluN2Amut)_2_ mediated currents; lower panels: (GluN1wt)_2_-(GluN2Bmut)_2_). **(C)** Summary of functional annotations of GluN2A/2B pairs. Data representing mean ± SEM. art, artificial GRIN2A variant; LBD, ligand binding domain; TMD, transmembrane domain; ns, non-significant statistical difference; *P < 0.05, **P < 0.01, ***P < 0.001, ****P < 0.0001; ND, not detectable; NA, not assigned.

Characterization of the biophysical behavior of mutant NMDARs was assessed in HEK-293T cells transiently expressing mutant di-heteromeric receptors. Similarly to the surface trafficking pattern effect between GluN2A-GluN2B equivalent pairs, whole-cell patch clamp experiments showed an overall conserved electrophysiological impact (Figures 2B,C, Supplementary Figure 3). The integration of putative biophysical parameters disturbances (normalized current amplitude, channel gating kinetics) induced by *GRIN2A*-*GRIN2B* variants pairs showed an overall coincident functional output in *GluN2A*-*GluN2B* variants pairs (Figure 2). In summary, the experimental evaluation of the structural algorithm predictive power showed the reliability of the model, in agreement with *in silico* comprehensive analysis of functionally annotated *GRIN* variants.

### 3.4 Application and predictive power of GRIN structural algorithm

**Figure 3:**
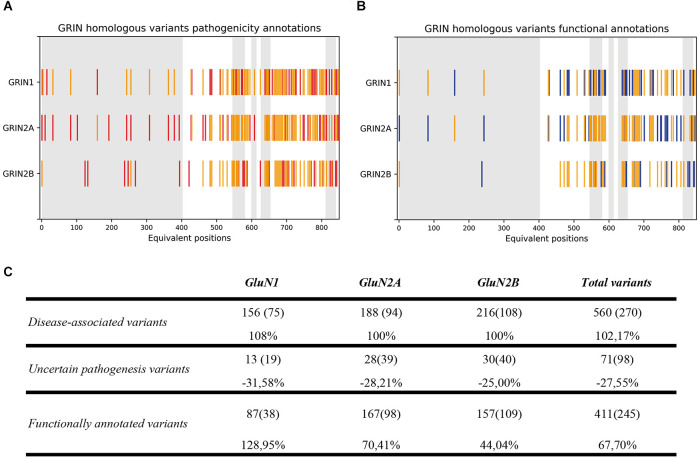
**(A)** GluN variants pathogenicity annotations based on current annotations (in red) and on GRIN structural algorithm-inferred annotations from homologous variants (in yellow). **(B)** GluN variants functional annotations based on current available annotations (in blue) and GRIN structural algorithm-inferred annotations from homologous variants (in yellow). **(C)** Summary of GRIN structural algorithm-based computational annotation of non-classified GluN1, GluN2A, and GluN2B subunits missense variants. GRIN predictive power of the algorithm was applied, and showed an expansion of disease-associated (top row) *GRIN* variants, together with a reduction of *GRIN* variants number previously classified as “Uncertain significance” (central row) and an increase of functional annotations of *GRIN* variants affecting the ATD, LBD, and TMD domains (bottom row).

Upon experimental and computational validation of the structural algorithm between GluN1, GluN2A, and GluN2B subunits, the predictive power of the GRIN structural algorithm was assessed. By applying the structural comparison algorithm to GRIN variants Database, 290 variants were predicted as pathogenic (representing a 100% increase in *GRIN* pathogenic variants), and 27 variants with uncertain significance were predicted as benign or pathogenic (representing a 28% decrease in non-stratified *GRIN1*, *GRIN2A*, *GRIN2B* missense variants; see Figure 3A). Concomitantly, functional annotations of 87 *GRIN1*, 167 *GRIN2A*, and 157 *GRIN2B* missense variants were predicted (representing a 68% increase for *GRIN1*, *GRIN2A*, and *GRIN2B* functional annotations; see Figure 3B). Figure 3C quantitatively summarizes the result of the GRIN structural algorithm application to GRINdb, showing both the increase in the number of *GRIN* pathogenic variants and in the classification as LoF/GoF/Complex using the *GRIN* structural algorithm.

### 3.5 GRIN structural algorithm model implementation into GRIN variants public database

Upon validation, the GRIN structural algorithm was implemented into GRIN variants Database^1^ (García-Recio et al.) This computational tool was adapted using a user-friendly interface, allowing the submission of individual *GRIN* variants queries, and retrieving the available disease-association and functional annotation based on homologous variants identified by means of the GRIN structural algorithm.

## 4 Discussion

Based on high-quality structural alignments of GluN1, GluN2A, and GluN2B ATD, LBD, we have generated the GRIN structural algorithm. This computational tool allows the identification of GluN subunit’s equivalent positions, in agreement with the high conservation degree of three-dimensional structure (vs. primary amino acid sequence conservation) in membrane proteins (Olivella et al., 2013). The equivalent positions in GluN subunits are predicted to maintain the same role in the structure and function of NMDA receptors. The algorithm predicts pathogenesis and functional annotations of non-annotated *GRIN* variants based on the annotations of homologous GluN variants, i.e., same amino acid change in the same equivalent position between GluN subunits. This model has been computationally evaluated in the bulk of previously annotated GluN1, GluN2A, and/or GluN2B missense variants pairs and trios from the major repertoire of annotations on *GRIN* variants. Further, the experimental validation of the algorithm, using *in silico*-designed *GRIN2A* variants putatively homologous to pathogenic *GRIN2B* variants has shown that pathogenesis and functional annotations can be extrapolated for homologous variants. Importantly, by using this structural alignment and identifying conserved equivalent positions, we have extended the pathogenesis and functional annotations of *GRIN1*, *GRIN2A*, and *GRIN2B* variants, duplicating the number of annotated pathogenic variants, decreasing by 27.5% the number of variants annotated with an uncertain significance through computational prediction performed by GRIN structural algorithm. Additionally, the number of functional annotations has increased by 67.7%, therefore narrowing the gap between genetically diagnosed *GRIN* pathogenic variants and their functional stratification. In addition to providing an acceleration of the most prevalent *GRIN* variants (affecting *GRIN1*, *GRIN2A*, and *GRIN2B* genes), the GRIN structural algorithm is expected to be refined and expanded by implementing future functional and structural studies. Compared to existing standard and non-specific gene variants predictors (i.e., SIFT, Polyphen-2), the hereby developed and validated GRIN structural algorithm provides a higher predictive power. Indeed, the aforementioned standard gene variants predictors have limited predictive power (accuracy estimated between 64% and 70%) for membrane proteins, including the NMDA receptor (García-Recio et al., 2021b). Additionally, these mutation predictors are not able to predict the functional impact of the mutation (loss-of-function, gain-of-function, or complex), which is of critical relevance in the context of GRIN-related disorders. Still, as functional annotations for homologous variants are not yet available for some GluN subunit positions, experimental validation and classic variant predictor tools are required.

Further, mutations affecting other *GRIN* genes should be considered. Noteworthy, *GRIN2D* gene variants might be potentially integrated into this model, upon the release of structural models. On the contrary, the application of the GRIN structural algorithm for *GRIN2C*, *GRIN3A*, and *GRIN3B* variants outcomes prediction will likely not be obvious. Indeed, genetic variants affecting these genes are often benign, suggesting that while the structural effects could be conserved with respect to other subunits (e.g., GluN1, GluN2A, GluN2B), their spatio-temporal expression pattern and/or the disturbance of their biophysical properties might result on the absence of clinical symptoms.

Beyond the direct interest for current and future *GRIN de novo* variants annotation and therapeutic advice, this innovative multidisciplinary computational/experimental approach could be extended to other channelopathies of primary genetic etiology, including but not limited to cardiopathies, skeletal muscle dystrophies, and metabolic disorders. Similarly to these conditions, disease-association and functional stratification of *GRIN* variants in GRD patients represent an important bottleneck to define the functional outcomes of non-annotated *GRIN* variants and envisioning and/or evaluating personalized therapies (Soto et al., 2019). In this context, although the GRIN structural algorithm is not sufficient *per se* to unequivocally drive the clinical decision, it provides an important investigational tool that will be nourished by future genetic, structural, and functional data, ultimately refining the functional annotation of *GRIN* variants.

## Data Availability Statement

The original contributions presented in the study are included in the article/Supplementary material, further inquiries can be directed to the corresponding author/s.

## Author Contributions

XA and MO conceived the study and designed the experiments and the computational analysis. XA, AS-G, and FM-C performed the experiments under the supervision of XA and DS. AG-R performed the computational analysis under the supervision of MO. XA and MO participated in the discussion and writing of the manuscript leading to the production of this article. All authors contributed to the article and approved the submitted version.

## Footnotes

^1^ http://lmc.uab.es/grindb

^2^ http://functionalvariants.emory.edu/database/

^3^ https://www.lovd.nl
