# Supplementary material for: Identification of homologous GluN subunits variants accelerates GRIN variants stratification
Source: Front Cell Neurosci. 2022 Dec 23;16:998719. doi: 10.3389/fncel.2022.998719 (PMC9816381; doi:10.3389/fncel.2022.998719)
Supplement: Supplementary file 1 [file Data_Sheet_1.PDF]

SUPPLEMENTARY MATERIAL

	GluN1		GluN2A		GluN2B	
	Start	*End*	*Start*	*End*	*Start*	*End*
**ATD**	1	393	1	401	1	402
**LBD**	394	548	402	543	403	544
	655	806	653	809	654	810
**TM**	549	654	544	652	545	653
	807	841	810	839	811	840

Supplementary Table 1. Residue ranges for each domain of the GluN1, GluN2A and GluN2B subunit domains.

Gene	Position	Initial amino acid	Final amino acid	Domain	Disease-association	Functional Classification
GRIN2A	32	Pro	Thr	ATD	Benign	
GRIN2B	32	Pro	Thr	ATD	Benign	
GRIN2A	32	Pro	Ser	ATD	Benign	
GRIN2B	32	Pro	Ser	ATD	Benign	
GRIN2A	45	Asp	Asn	ATD	Benign	
GRIN2B	46	Asp	Asn	ATD	Benign	
GRIN2A	90	Met	Thr	ATD	Benign	
GRIN2B	89	Met	Thr	ATD	Benign	
GRIN2A	212	Asp	Asn	ATD	Benign	
GRIN2B	211	Asp	Asn	ATD	Benign	
GRIN1	219	Ile	Val	ATD	Benign	
GRIN2B	227	Ile	Val	ATD	Benign	
GRIN2A	272	Phe	Leu	ATD	Benign	
GRIN2B	273	Phe	Leu	ATD	Benign	
GRIN2A	279	Val	Ile	ATD	Benign	
GRIN2B	280	Val	Ile	ATD	Benign	
GRIN2A	298	Ile	Val	ATD	Benign	
GRIN2B	299	Ile	Val	ATD	Benign	
GRIN1	364	Val	Met	ATD	Benign	
GRIN2A	375	Val	Met	ATD	Benign	

GRIN2A	424	Pro	Arg	LBD	Benign	
GRIN2B	424	Pro	Arg	LBD	Benign	
GRIN2A	436	Cys	Arg	LBD	Pathogenic	LoF
GRIN2B	436	Cys	Arg	LBD	Pathogenic	LoF
GRIN2A	448	Glu	Lys	LBD	Benign	
GRIN2B	448	Glu	Lys	LBD	Benign	
GRIN2A	455	Cys	Tyr	LBD	Pathogenic	
GRIN2B	456	Cys	Tyr	LBD	Pathogenic	LoF
GRIN2A	458	Gly	Glu	LBD	Pathogenic	
GRIN2B	459	Gly	Glu	LBD	Pathogenic	
GRIN1	523	Arg	Cys	LBD	Pathogenic	LoF
GRIN2A	518	Arg	Cys	LBD	Pathogenic	LoF
GRIN2A	532	Gly	Val	LBD	Pathogenic	
GRIN2B	533	Gly	Val	LBD	Pathogenic	
GRIN1	557	Pro	Arg	TMD	Pathogenic	Complex
GRIN2A	552	Pro	Arg	TMD	Pathogenic	Complex
GRIN1	557	Pro	Leu	TMD	Pathogenic	
GRIN2B	553	Pro	Leu	TMD	Pathogenic	LoF
GRIN1	616	Asn	Ser	TMD	Pathogenic	
GRIN2A	614	Asn	Ser	TMD	Pathogenic	LoF
GRIN2A	615	Asn	Lys	TMD	Pathogenic	Complex
GRIN2B	616	Asn	Lys	TMD	Pathogenic	Complex
GRIN1	637	Ala	Val	TMD	Pathogenic	GoF
GRIN2B	636	Ala	Val	TMD	Pathogenic	GoF
GRIN2A	638	Ala	Val	TMD	Pathogenic	
GRIN2B	639	Ala	Val	TMD	Pathogenic	GoF
GRIN2A	648	Asn	Ser	TMD	Pathogenic	
GRIN2B	649	Asn	Ser	TMD	Pathogenic	GoF
GRIN2B	649	Asn	Thr	TMD	Pathogenic	GoF
GRIN1	650	Asn	Thr	TMD	Pathogenic	
GRIN1	653	Ala	Gly	TMD	Pathogenic	GoF
GRIN2B	652	Ala	Gly	TMD	Pathogenic	
GRIN2A	694	Ile	Thr	LBD	Pathogenic	LoF
GRIN2B	695	Ile	Thr	LBD	Pathogenic	LoF
GRIN1	706	Met	Val	LBD	Pathogenic	

GRIN2A	705	Met	Val	LBD	Pathogenic	LoF
GRIN2B	706	Met	Val	LBD	Pathogenic	
GRIN2B	732	Asp	Glu	LBD	Pathogenic	
GRIN1	732	Asp	Glu	LBD	Pathogenic	LoF
GRIN2A	742	Asp	Glu	LBD	Benign	
GRIN2B	743	Asp	Glu	LBD	Benign	
GRIN1	744	Cys	Tyr	LBD	Pathogenic	GoF
GRIN2B	746	Cys	Tyr	LBD	Pathogenic	
GRIN2A	749	Thr	Ile	LBD	Pathogenic	
GRIN2B	750	Thr	Ile	LBD	Pathogenic	
GRIN2A	809	Ser	Arg	LBD	Pathogenic	GoF
GRIN2B	810	Ser	Arg	LBD	Pathogenic	GoF
GRIN2A	817	Met	Thr	TMD	Pathogenic	
GRIN2B	818	Met	Thr	TMD	Pathogenic	GoF
GRIN1	813	Met	Thr	TMD	Pathogenic	
GRIN2B	820	Gly	Arg	TMD	Pathogenic	
GRIN1	815	Gly	Arg	TMD	Pathogenic	Complex
GRIN1	815	Gly	Val	TMD	Pathogenic	LoF
GRIN2B	820	Gly	Val	TMD	Pathogenic	LoF
GRIN2B	820	Gly	Ala	TMD	Pathogenic	LoF
GRIN2A	819	Gly	Ala	TMD	Pathogenic	
GRIN2A	839	His	Gln	TMD	Benign	
GRIN2B	840	His	Gln	TMD	Benign	

Supplementary Table 2. Disease-association and functional classification of structural homologous variants pairs and trios, extracted from reported GRIN variants compiled in GRIN variants Database. *LoF*, loss-of-function variants. *GoF*, gain-of-function variants. Pathogenic variants are "pathogenic, strong" and benign variants are "benign, strong" according to ACGM classification guidance.

**Gene**	**Position**	**Initial amino acid**	**Final amino acid**	**Domain**	**Disease-association**	**Functional Classification**
GRIN1	5	Arg	Ser	ATD	Benign	
GRIN2A	5	Gly	Ser	ATD	Benign	
GRIN2A	13	Pro	Leu	ATD	Benign	
GRIN2B	13	Trp	Leu	ATD	Benign	
GRIN1	205	Thr	Met	ATD	Benign	

GRIN2B	216	Ile	Met	ATD	Benign	
GRIN1	416	Ser	Arg	ATD	Benign	
GRIN2A	424	Pro	Arg	ATD	Benign	
GRIN2B	424	Pro	Arg	ATD	Benign	
GRIN1	424	Phe	Ser	LBD	Benign	
GRIN2A	432	Asn	Ser	LBD	Benign	
GRIN1	440	Asn	Thr	LBD	Benign	
GRIN2B	440	Ile	Thr	LBD	Benign	
GRIN1	461	Asp	Val	LBD	Benign	
GRIN2B	462	Ile	Val	LBD	Benign	
GRIN1	559	Gln	Arg	TMD	Pathogenic	
GRIN2A	554	Ser	Arg	TMD	Pathogenic	
GRIN2A	571	Ile	Met	TMD	Benign	
GRIN2B	572	Val	Met	TMD	Benign	
GRIN1	593	Ser	Thr	IC1	Benign	
GRIN2B	590	Ala	Thr	IC1	Benign	
GRIN1	641	Met	Ile	TMD	Pathogenic	GoF
GRIN2A	639	Val	Ile	TMD	Pathogenic	GoF
GRIN1	644	Val	Met	TMD	Pathogenic	
GRIN2A	642	Leu	Met	TMD	Pathogenic	GoF
GRIN1	685	Lys	Arg	LBD	Benign	
GRIN2B	687	Pro	Arg	LBD	Pathogenic	LoF

Supplementary Table 3. Disease-association and functional classification of structural equivalent variants with different initial amino acid and identical final amino acid, extracted from GRIN variants Database. Pathogenic variants are "pathogenic, strong" and benign variants are "benign, strong" according to ACGM classification guidance. *LoF*, loss-of-function variants. *GoF*, gain-of-function variants.

Gene	Amino acid position	Reference Amino acid		Domain	Disease-association	Gain/Loss
GRIN1	5	Arg	His	ATD	Benign	
GRIN1	5	Arg	Ser	ATD	Benign	
GRIN2A	8	Thr	Ile	ATD	Benign	
GRIN1	8	Thr	Lys	ATD	Benign	
GRIN1	8	Thr	Pro	ATD	Benign	
GRIN2B	26	Ala	Gly	ATD	Benign	

GRIN2A	26	Ala	Ser	ATD	Benign
GRIN2A	26	Ala	Val	ATD	Benign
GRIN2A	31	Pro	Ser	ATD	Benign
GRIN2B	32	Pro	Ser	ATD	Benign
GRIN2A	31	Pro	Thr	ATD	Pathogenic
GRIN2B	32	Pro	Thr	ATD	Benign
GRIN2B	33	Pro	Arg	ATD	Benign
GRIN2A	32	Pro	Ser	ATD	Benign
GRIN2A	40	Leu	Met	ATD	Benign
GRIN1	33	Leu	Val	ATD	Benign
GRIN2A	90	Met	Ile	ATD	Benign
GRIN2A	90	Met	Thr	ATD	Benign
GRIN2B	89	Met	Thr	ATD	Benign
GRIN2B	132	Met	Ile	ATD	Benign
GRIN2A	133	Met	Val	ATD	Benign
GRIN2B	229	Leu	Ile	ATD	Benign
GRIN2A	228	Leu	Phe	ATD	Benign
GRIN1	235	Ala	Pro	ATD	Benign
GRIN2B	244	Ala	Ser	ATD	Benign
GRIN2A	243	Ala	Val	ATD	Pathogenic
GRIN2B	266	Thr	Ile	ATD	Benign
GRIN2A	265	Thr	Met	ATD	Benign
GRIN2A	298	Ile	Val	ATD	Benign
GRIN2B	299	Ile	Val	ATD	Benign
GRIN2B	312	Ser	Asn	ATD	Benign
GRIN2A	311	Ser	Tyr	ATD	Benign
GRIN2A	319	Ser	Arg	ATD	Benign
GRIN2A	319	Ser	Asn	ATD	Benign
GRIN2B	320	Ser	Thr	ATD	Benign
GRIN2B	359	His	Asn	ATD	Benign
GRIN2A	358	His	Gln	ATD	Benign
GRIN2A	358	His	Leu	ATD	Benign
GRIN2A	367	Asn	Lys	ATD	Benign
GRIN2B	368	Asn	Thr	ATD	Benign
GRIN1	359	Arg	His	ATD	Benign
GRIN2B	371	Arg	Met	ATD	Benign

GRIN2A	424	Pro	Arg	LBD	Benign	
GRIN2B	424	Pro	Arg	LBD	Benign	
GRIN2A	424	Pro	His	LBD	Benign	
GRIN2A	424	Pro	Leu	LBD	Benign	
GRIN2A	436	Cys	Arg	LBD	Pathogenic	LoF
GRIN2B	436	Cys	Arg	LBD	Pathogenic	LoF
GRIN2A	436	Cys	Phe	LBD	Pathogenic	
GRIN2A	440	Val	Ile	LBD	Benign	
GRIN2A	440	Val	Leu	LBD	Benign	
GRIN2B	442	Thr	Ala	LBD	Benign	
GRIN1	442	Thr	Met	LBD	Benign	
GRIN2A	444	Asn	Ser	LBD	Benign	
GRIN2B	444	Asn	Tyr	LBD	Benign	
GRIN1	447	Pro	His	LBD	Benign	
GRIN1	447	Pro	Leu	LBD	Benign	
GRIN1	447	Pro	Ser	LBD	Benign	
GRIN2A	448	Glu	Asp	LBD	Benign	
GRIN2A	448	Glu	Lys	LBD	Benign	
GRIN2B	448	Glu	Lys	LBD	Benign	
GRIN2B	459	Gly	Arg	LBD	Pathogenic	LoF
GRIN2A	458	Gly	Glu	LBD	Pathogenic	
GRIN2B	459	Gly	Glu	LBD	Pathogenic	
GRIN1	457	Gly	Ser	LBD	Benign	
GRIN2A	462	Asp	Glu	LBD	Benign	
GRIN1	461	Asp	Gly	LBD	Benign	
GRIN1	461	Asp	Val	LBD	Benign	
GRIN2A	483	Gly	Arg	LBD	Pathogenic	LoF
GRIN2B	484	Gly	Asp	LBD	Pathogenic	LoF
GRIN2A	498	Gly	Ala	LBD	Benign	
GRIN2B	499	Gly	Arg	LBD	Pathogenic	LoF
GRIN2A	498	Gly	Ser	LBD	Pathogenic	
GRIN2B	514	Thr	Ala	LBD	Pathogenic	LoF
GRIN2A	513	Thr	Ile	LBD	Pathogenic	
GRIN1	523	Arg	Cys	LBD	Pathogenic	LoF
GRIN2A	518	Arg	Cys	LBD	Pathogenic	LoF
GRIN2B	519	Arg	Gln	LBD	Pathogenic	

GRIN2B	519	Arg	Gly	LBD	Pathogenic	LoF
GRIN2A	518	Arg	His	LBD	Pathogenic	LoF
GRIN2A	518	Arg	Leu	LBD	Pathogenic	
GRIN2A	527	Pro	Arg	LBD	Pathogenic	LoF
GRIN1	532	Pro	His	LBD	Pathogenic	LoF
GRIN1	532	Pro	Leu	LBD	Pathogenic	
GRIN2B	532	Thr	Ala	LBD	Pathogenic	LoF
GRIN2A	531	Thr	Met	LBD	Pathogenic	LoF
GRIN2B	533	Gly	Asp	LBD	Pathogenic	LoF
GRIN2A	532	Gly	Val	LBD	Pathogenic	
GRIN2B	533	Gly	Val	LBD	Pathogenic	
GRIN2A	548	Ala	Pro	TMD	Pathogenic	
GRIN2A	548	Ala	Thr	TMD	Pathogenic	LoF
GRIN2B	549	Ala	Val	TMD	Pathogenic	LoF
GRIN1	557	Pro	Arg	TMD	Pathogenic	LoF
GRIN2A	552	Pro	Arg	TMD	Pathogenic	GoF
GRIN1	557	Pro	Leu	TMD	Pathogenic	
GRIN2B	553	Pro	Leu	TMD	Pathogenic	LoF
GRIN2B	553	Pro	Thr	TMD	Pathogenic	LoF
GRIN2A	554	Ser	Arg	TMD	Pathogenic	
GRIN2B	555	Ser	Asn	TMD	Pathogenic	LoF
GRIN2B	555	Ser	Ile	TMD	Pathogenic	
GRIN2B	559	Trp	Arg	TMD	Pathogenic	LoF
GRIN2A	558	Trp	Ser	TMD	Pathogenic	
GRIN2A	589	Ala	Gly	IC1	Benign	
GRIN2A	589	Ala	Ser	IC1	Benign	
GRIN2B	590	Ala	Thr	IC1	Benign	
GRIN2B	595	Pro	Ala	IC1	Benign	
GRIN2A	594	Pro	Leu	IC1	Benign	
GRIN2A	594	Pro	Ser	IC1	Benign	
GRIN2B	607	Trp	Cys	TMD	Pathogenic	LoF
GRIN2B	607	Trp	Ser	TMD	Pathogenic	GoF
GRIN2B	615	Asn	Ile	TMD	Pathogenic	LoF
GRIN2B	615	Asn	Lys	TMD	Pathogenic	LoF
GRIN1	616	Asn	Ser	TMD	Pathogenic	
GRIN2A	614	Asn	Ser	ML	Pathogenic	LoF

GRIN2A	615	Asn	Lys	ML	Pathogenic	Complex
GRIN2B	616	Asn	Lys	ML	Pathogenic	Complex
GRIN2A	615	Asn	Ser	ML	Pathogenic	GoF
GRIN2B	628	Ser	Phe	M3	Pathogenic	LoF
GRIN2B	628	Ser	Tyr	M3	Pathogenic	LoF
GRIN2B	636	Ala	Pro	M3	Pathogenic	LoF
GRIN2A	635	Ala	Thr	M3	Pathogenic	GoF
GRIN1	637	Ala	Val	M3	Pathogenic	GoF
GRIN2B	636	Ala	Val	M3	Pathogenic	GoF
GRIN2B	639	Ala	Ser	M3	Benign	
GRIN2B	639	Ala	Val	M3	Pathogenic	GoF
GRIN1	642	Ile	Leu	M3	Pathogenic	
GRIN2B	641	Ile	Thr	M3	Pathogenic	
GRIN2A	642	Leu	Met	M3	Pathogenic	GoF
GRIN2B	643	Leu	Pro	M3	Pathogenic	
GRIN2A	643	Ala	Asp	M3	Pathogenic	GoF
GRIN1	645	Ala	Ser	M3	Pathogenic	LoF
GRIN1	647	Tyr	Cys	M3	Pathogenic	Complex
GRIN1	647	Tyr	Ser	M3	Pathogenic	LoF
GRIN1	650	Asn	Ile	M3	Pathogenic	GoF
GRIN1	650	Asn	Lys	M3	Pathogenic	
GRIN2A	648	Asn	Ser	M3	Pathogenic	
GRIN2B	649	Asn	Ser	M3	Pathogenic	GoF
GRIN2B	649	Asn	Thr	M3	Pathogenic	GoF
GRIN1	650	Asn	Thr	M3	Pathogenic	
GRIN2A	649	Leu	Pro	M3	Pathogenic	
GRIN2A	649	Leu	Val	M3	Pathogenic	GoF
GRIN1	653	Ala	Gly	M3	Pathogenic	GoF
GRIN2B	652	Ala	Gly	M3	Pathogenic	
GRIN2B	652	Ala	Pro	M3	Pathogenic	LoF
GRIN1	654	Phe	Cys	M3	Pathogenic	GoF
GRIN2A	652	Phe	Val	M3	Pathogenic	GoF
GRIN2B	655	Ile	Phe	LBD	Pathogenic	GoF
GRIN2A	654	Ile	Thr	LBD	Pathogenic	GoF
GRIN2A	675	Asp	Ala	LBD	Benign	
GRIN2B	676	Asp	Asn	LBD	Pathogenic	

GRIN2A	675	Asp	Glu	LBD	Benign	
GRIN2A	681	Arg	Gln	LBD	Benign	
GRIN2B	682	Arg	His	LBD	Pathogenic	
GRIN1	688	Ser	Pro	LBD	Pathogenic	
GRIN1	688	Ser	Tyr	LBD	Pathogenic	
GRIN2B	691	Thr	Ile	LBD	Pathogenic	LoF
GRIN2A	690	Thr	Met	LBD	Pathogenic	LoF
GRIN2B	695	Ile	Ser	LBD	Pathogenic	
GRIN2A	694	Ile	Thr	LBD	Pathogenic	LoF
GRIN2B	695	Ile	Thr	LBD	Pathogenic	LoF
GRIN2A	695	Arg	Gln	LBD	Pathogenic	GoF
GRIN2B	696	Arg	His	LBD	Pathogenic	GoF
GRIN1	694	Arg	Pro	LBD	Benign	
GRIN2B	696	Arg	Ser	LBD	Pathogenic	
GRIN2A	731	Asp	Asn	LBD	Pathogenic	LoF
GRIN1	732	Asp	Glu	LBD	Pathogenic	LoF
GRIN2B	732	Asp	Glu	LBD	Pathogenic	
GRIN2A	733	Ala	Thr	LBD	Pathogenic	LoF
GRIN2B	734	Ala	Val	LBD	Pathogenic	LoF
GRIN2B	751	Ile	Leu	LBD	Pathogenic	LoF
GRIN2B	751	Ile	Thr	LBD	Pathogenic	LoF
GRIN2A	750	Ile	Val	LBD	Benign	
GRIN2A	753	Gly	Ala	LBD	Pathogenic	
GRIN2B	754	Gly	Arg	LBD	Benign	
GRIN1	772	Val	Ile	LBD	Benign	
GRIN1	772	Val	Leu	LBD	Benign	
GRIN2A	780	Leu	Trp	LBD	Pathogenic	
GRIN2B	781	Leu	Val	LBD	Pathogenic	LoF
GRIN2A	788	Met	Ile	LBD	Benign	
GRIN2B	789	Met	Lys	LBD	Pathogenic	
GRIN2A	809	Ser	Arg	LBD	Pathogenic	GoF
GRIN2B	810	Ser	Arg	LBD	Pathogenic	GoF
GRIN2B	810	Ser	Asn	LBD	Pathogenic	GoF
GRIN2A	817	Met	Arg	TMD	Pathogenic	
GRIN2B	818	Met	Leu	TMD	Pathogenic	
GRIN2A	817	Met	Thr	TMD	Pathogenic	

GRIN2B	818	Met	Thr	TMD	Pathogenic	GoF
GRIN2A	817	Met	Val	TMD	Pathogenic	GoF
GRIN1	814	Ala	Asp	TMD	Pathogenic	
GRIN2A	818	Ala	Glu	TMD	Pathogenic	GoF
GRIN2B	819	Ala	Thr	TMD	Pathogenic	GoF
GRIN2A	818	Ala	Val	TMD	Benign	
GRIN2B	820	Gly	Ala	TMD	Pathogenic	LoF
GRIN2A	819	Gly	Ala	TMD	Pathogenic	
GRIN1	815	Gly	Arg	TMD	Pathogenic	LoF
GRIN2B	820	Gly	Arg	TMD	Pathogenic	
GRIN2B	820	Gly	Glu	TMD	Pathogenic	LoF
GRIN1	815	Gly	Val	TMD	Pathogenic	LoF
GRIN2B	820	Gly	Val	TMD	Pathogenic	LoF
GRIN2A	820	Val	Gly	TMD	Benign	
GRIN2B	821	Val	Phe	TMD	Pathogenic	LoF
GRIN2B	824	Met	Arg	TMD	Pathogenic	LoF
GRIN2A	823	Met	Ile	TMD	Pathogenic	
GRIN2B	824	Met	Val	TMD	Pathogenic	LoF
GRIN2A	836	Ile	Leu	TMD	Benign	
GRIN2A	836	Ile	Thr	TMD	Benign	
GRIN2A	839	His	Gln	TMD	Benign	
GRIN2B	840	His	Gln	TMD	Benign	
GRIN2A	839	His	Tyr	TMD	Benign	

Supplementary Table 4. Disease-association and functional classification of structural equivalent variants pairs and trios with same initial amino acid and different final amino acid, extracted from *GRIN* variants Database. Pathogenic variants are "pathogenic, strong" and benign variants are "benign, strong" according to ACGM classification guidance. *LoF*, loss-of-function variants. *GoF*, gain-of-function variants.

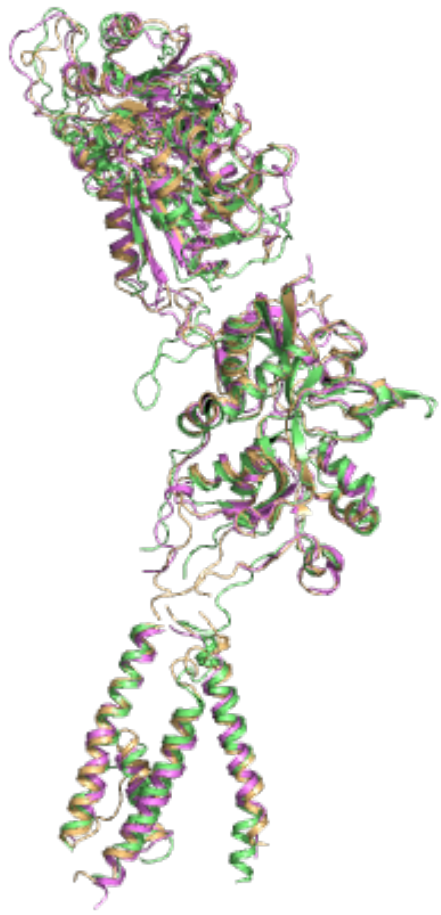

Supplementary Figure 1. Structural superimposition of the GluN1 (in green), GluN2A (in pink) and GluN2B (in orange) subunits domains of the NMDA receptor. The structural superimposition model allows to identify equivalent positions across GluN subunits.

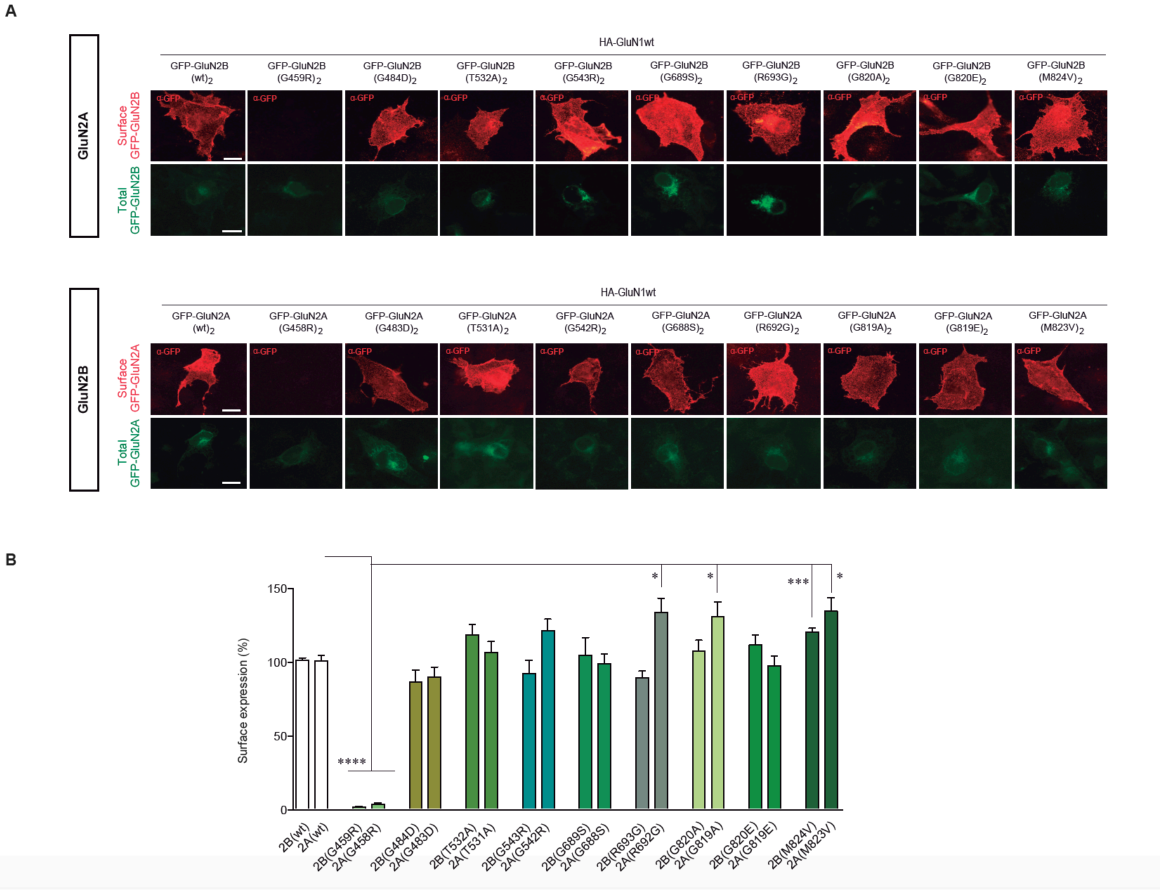

Supplementary Figure 2: ***A***, Immunofluorescence analysis of NMDAR surface expression in COS-7 cells transiently transfected with GFP-Grin2b (wild-type or mutant) or GFP-Grin2a (wild-type or mutant) and HA-Grin1 wild-type constructs; scale bar: 10 µm; **B**, Bar graphs representing the mean ± SEM of cell surface expression of NMDAR (N = > 20 cells per condition, from at least 3 independent experiments; ns, P-value > 0.05; *, P-value < 0.01; ***, P-value < 0.001; ****, P-value < 0.0001, one-way ANOVA with Bonferroni's post hoc test).

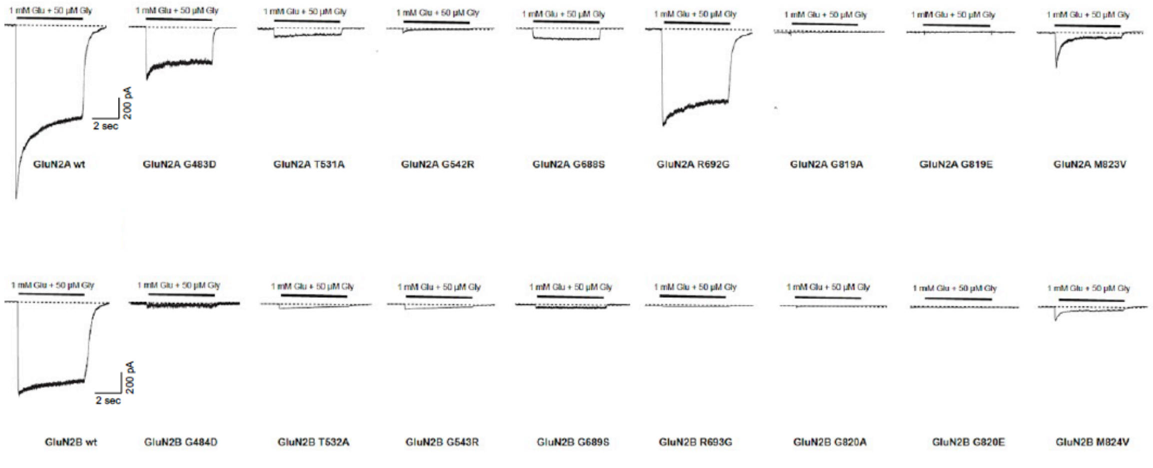

Supplementary Figure 3. Functional annotation of GluN2A and GluN2B variants. (A) Representative whole-cell currents evoked by rapid application of 1 mM glutamate plus 50 µM glycine (0.5-second duration; −60 mV) in HEK-293T cells expressing $(GluN1wt)_2$-(GluN2Awt/mut) (top traces) $(GluN1wt)_2$-(GluN2Bwt/mut) (bottom traces).
